# *TP53* codon 72 polymorphism is associated with *FGFR3* and *RAS* mutation in non-muscle-invasive bladder cancer

**DOI:** 10.1371/journal.pone.0220173

**Published:** 2019-08-01

**Authors:** Takashi Kawahara, Takahiro Kojima, Shuya Kandori, Masahiro Kurobe, Takayuki Yoshino, Tomokazu Kimura, Yoshiyuki Nagumo, Ryutaro Ishituka, Koji Mitsuzuka, Shintaro Narita, Takashi Kobayashi, Yoshiyuki Matsui, Osamu Ogawa, Mikio Sugimoto, Jun Miyazaki, Hiroyuki Nishiyama

**Affiliations:** 1 Department of Urology, Faculty of Medicine, University of Tsukuba, Tsukuba, Japan; 2 Department of Urology, Graduate School of Medicine, Tohoku University, Sendai, Japan; 3 Department of Urology, Graduate School of Medicine, Akita University, Akita, Japan; 4 Department of Urology, Graduate School of Medicine, Kyoto University, Kyoto, Japan; 5 Department of Urology, Faculty of Medicine, Kagawa University, Kagawa, Japan; 6 Department of Urology, School of Medicine, International University of Health and Welfare, Narita, Japan; National Cancer Center, JAPAN

## Abstract

**Objective:**

*TP53*, a well-known tumor-suppressor gene in bladder carcinogenesis, has a functional single-nucleotide polymorphism on codon 72. The aim of this study was to elucidate the association between *TP53* codon 72 polymorphism and somatic mutations in bladder cancer.

**Material and methods:**

Germline *TP53* codon 72 polymorphism and somatic mutations of 50 cancer-associated genes were analyzed in 103 bladder cancer patients (59 non-muscle-invasive and 44 muscle-invasive), using Taqman genotyping assay and target sequencing, respectively. The expression of FGF-FGFR signaling pathway genes was analyzed by RNA sequencing of frozen tissue.

**Results:**

The allele frequency of *TP53* codon 72 in our cohort was 37, 42, and 21% for Arg/Arg, Arg/Pro, and Pro/Pro, respectively. Interestingly, the prevalence of *FGFR3* mutation was higher in patients with the Arg allele, whereas that of the *RAS* mutation was higher in patients without the Arg allele. The same association was seen in non-muscle-invasive bladder cancer (NMIBC) patients and no differences were observed in muscle-invasive bladder cancer patients. In NMIBC, *FGFR1* expression was higher in patients without the Arg allele and *FGFR3* expression was higher in patients with the Arg allele.

**Conclusion:**

The germline *TP53* codon 72 polymorphism was associated with mutations of *FGFR3* or *RAS* and expression of *FGFR1* and *FGFR3* in NMIBC. These findings provide new insight into the molecular mechanisms underlying the influence of the genetic background on carcinogenesis in bladder cancer.

## Introduction

Bladder cancer is the fourth and twelfth most common malignancy in men and women, respectively [1]. It is particularly common in the elderly and male population. Cigarette smoking and some Chinese herbs are well-known risk factors [2–5]. Bladder cancer is derived from urothelium and progresses from non-muscle-invasive bladder cancer (NMIBC) to muscle-invasive bladder cancer (MIBC), before becoming metastatic. About 70% of bladder cancer patients have NMIBC, and the remaining 30% have MIBC or metastatic disease [6]. Molecular and histopathological features suggest that bladder cancer can develop along at least two distinct pathways. In one pathway, the papillary NMIBC develop via epithelial hyperplasia and recruitment of branching vasculature, and in the other pathway MIBC develops via flat dysplasia and carcinoma *in situ* (CIS) [2, 7]. Several important driver genes or tumor suppressor genes involved in carcinogenesis of bladder urothelium have been identified. For example, *TP53* mutations are key drivers for CIS or MIBC [8]. On the other hand, NMIBC is characterized by activating point mutations in *FGFR3* or *RAS* [9, 10], and, interestingly, activating *RAS* mutations are mutually exclusive with *FGFR3* mutations [10]. However, the underlying mechanism controlling the selection of specific somatic mutations in bladder cancer remains unknown.

TP53 functions as a transcription factor, regulating the expression of several downstream genes, resulting in cell cycle arrest and apoptosis [11]. *TP53* is also known to have a functional single-nucleotide polymorphism (SNP) in codon 72 (rs1042522), which results in the substitution of proline (Pro) for arginine (Arg) in the proline-rich domain. *TP53* Arg72 is more potent in apoptosis induction, whereas *TP53* Pro72 is better in inducing cell cycle arrest and DNA damage repair [12–15]. *TP53* codon 72 polymorphism has been linked to an increased risk of breast cancer, cervical cancer, esophageal cancer, gastric cancer, lung cancer, and skin cancer [16]. However, studies relating to the association between *TP53* codon 72 polymorphism and bladder cancer susceptibility have shown inconclusive results [16]. We previously reported that this polymorphism affects the phenotypes or clinical outcomes of bladder cancer [17], but the underlying mechanism remains unknown.

Recently, several studies reported the interesting finding that germline SNPs affect specific somatic mutations. For instance, MC1R polymorphism affects *BRAF* mutant melanoma [18, 19], a *JAK2* germline polymorphism affects *JAK2* V617F mutant myeloproliferative neoplasms [20, 21], and *TACC3* polymorphism affects *FGFR3* mutant bladder cancer [22]. Some reports showed that the *TP53* Pro allele is associated with an increased frequency of *TP53* mutations in non-small cell lung cancer (NSCLC) [23, 24]. However, there is no report around the relationship between *TP53* codon 72 polymorphism and somatic mutations in bladder cancer.

We hypothesized that *TP53* codon 72 polymorphism could affect somatic mutations during bladder carcinogenesis and conducted this study to compare germline *TP53* codon 72 polymorphism and somatic mutations in bladder cancer. In our cohort, there was no relationship between *TP53* codon 72 polymorphism and *TP53* mutation. However, mutually exclusive mutations of *FGFR3* and *RAS* in NMIBC were significantly related with the *TP53* codon 72 polymorphism. This finding provides new insight into the relationship between host germline polymorphism and selection for somatic mutation type in bladder carcinogenesis.

## Materials and methods

### Patients and tissue samples

This prospective multicenter cohort study included 144 patients with clinical diagnosis of urothelial carcinoma from seven institutions [25]. The research protocol was approved by the Ethics Committee of Tsukuba University Hospital (Approval number: H25-116). It was also reviewed and approved by the Ethics Committees of the following institutes: Tohoku University Hospital, Akita University Hospital, Kyoto University Hospital, Kagawa University Hospital, Hitachi General Hospital, and Tsukuba Medical Center Hospital. Tumor specimens, blood, and clinicopathological information were collected with written informed consent.

Primary bladder cancer tissue samples from 103 patients were stored as formalin-fixed paraffin embedded (FFPE) and frozen tissue. All tissue sections included malignant tumor cell nuclei in 10% or more cells of the whole specimen. The remaining 41 patients were excluded because their tumors originated in the upper urinary tract origin, were without urothelial histology, or fresh frozen tissue could not be obtained.

Hematoxylin and eosin staining was performed, and the slides were evaluated by pathologists at each institute. Tumors were staged according to the 2009 Union for International Cancer Control (UICC) 7th tumor-nodes-metastasis (TNM) classification system.

### Tumor DNA extraction from FFPE samples and mutation analysis

Tumor DNA from FFPE was extracted using a QIAamp DNA FFPE Tissue Kit (QIAGEN, Hilden, Limburg, Germany) according to the manufacturer’s instructions. The DNA concentration was assessed using a Qubit fluorometer (Thermo Fisher Scientific, Waltham, MA, USA). Tumor DNA with more than 1.5 ng/μL, according to the Qubit fluorometer, was subjected to further analysis. In total, 10 ng DNA was used as template to generate an amplicon library for sequencing. Libraries were prepared using an Ion AmpliSeq Library Kit 2.0 and an Ion AmpliSeq Cancer Hotspot Panel v2 (Life Technologies, Waltham, MA, USA), which amplifies 207 amplicons covering approximately 2800 COSMIC mutations in the following 50 cancer-associated genes in alphabetical order: *ABL1*, *AKT1*, *ALK*, *APC*, *ATM*, *BRAF*, *CDH1*, *CDKN2A*, *CSF1R*, *CTNNB1*, *EGFR*, *ERBB2*, *ERBB4*, *EZH2*, *FBXW7*, *FGFR1*, *FGFR2*, *FGFR3*, *FLT3*, *FRBB4*, *GNA11*, *GNAS*, *HNF1A*, *HRAS*, *IDH1*, *IDH2*, *JAK2*, *JAK3*, *KDR*, *KIT*, *KRAS*, *MET*, *MLH1*, *MPL*, *NOTCH1*, *NPM1*, *NRAS*, *PDGFRA*, *PIK3CA*, *PTEN*, *PTPN11*, *RB1*, *RET*, *SMAD4*, *SMARCB1*, *SMO*, *SRC*, *STK11*, *TP53*, *VHL*. Adapter ligation, nick repair, and polymerase chain reaction (PCR) amplification were performed according to the manufacturer's instructions. Emulsion PCR and enrichment steps were carried out using an Ion OneTouch Template Kit and an Ion OneTouch system (Life Technologies), according to the manufacturer's instructions. Following enrichment, the amplicon libraries were sequenced with an Ion PGM Sequencer (Life Technologies). For data analysis, Torrent Suite 4.0.2 was used, and mutations were detected by the Variant Caller plugin 4.0–6 with somatic/high stringency configuration provided by Ion Torrent (Thermo Fisher Scientific).

### Germline DNA extraction and *TP53* codon 72 genotyping

Germline DNA was extracted from peripheral blood using the DNeasy Blood and Tissue Kit (QIAGEN) according to the manufacturer’s instructions. Germline DNA samples were genotyped using TaqMan single-nucleotide polymorphism genotyping assays for rs1042522 (Applied Biosystems, Waltham, MA, USA) according to the manufacturer’s instructions. The results were analyzed on a 7500 real-time PCR system using the allelic discrimination assay program of Sequence Detection software version 1.3 (Applied Biosystems).

### RNA extraction and sequence analysis

Total RNA was extracted from frozen tissue using TRIzol (Thermo Fisher Scientific), prepared into messenger RNA (mRNA) libraries, and sequenced using Illumina NextSeq 500. Quality control, ambiguity and length trimming, mapping to the reference genome, normalization of gene expression, and evaluation of differential gene expression were performed using CLC Genomics Workbench version 10 (Qiagen). Default settings were used for quality control and ambiguity and length trimming. RNA-sequence reads were aligned to the reference genome of *Homo sapiens* GRCh38.p10 (GenBank accession number GCA_000001405.25).

### Statistical analysis

Differences were assessed using Kruskal-Wallis test and Mann-Whitney U test with Bonferroni correction. The chi-square test was used to evaluate associations between categorical variables. When the *p*-value was *p* < 0.05 with chi-square test, residual analysis was performed to identify which category was significant. Gene expression was normalized using transcript per million. Wald test with Benjamini-Hochberg multiple test correction was used for evaluation of differential gene expression. Genes with false discovery rate adjusted *p-*values < 0.05 were considered differentially expressed. Adjusted residuals were calculated with js-STAR ver 9.1.7 [26], evaluation of differential gene expression was performed using CLC Genomics Workbench version 10 (Qiagen), and the other statistical analyses were performed using STAT view ver5.0 (SAS Institute Inc. Cary, NC, USA).

## Results

### Association between germline *TP53* codon 72 polymorphism and somatic mutations in bladder cancer

**Fig 1:**
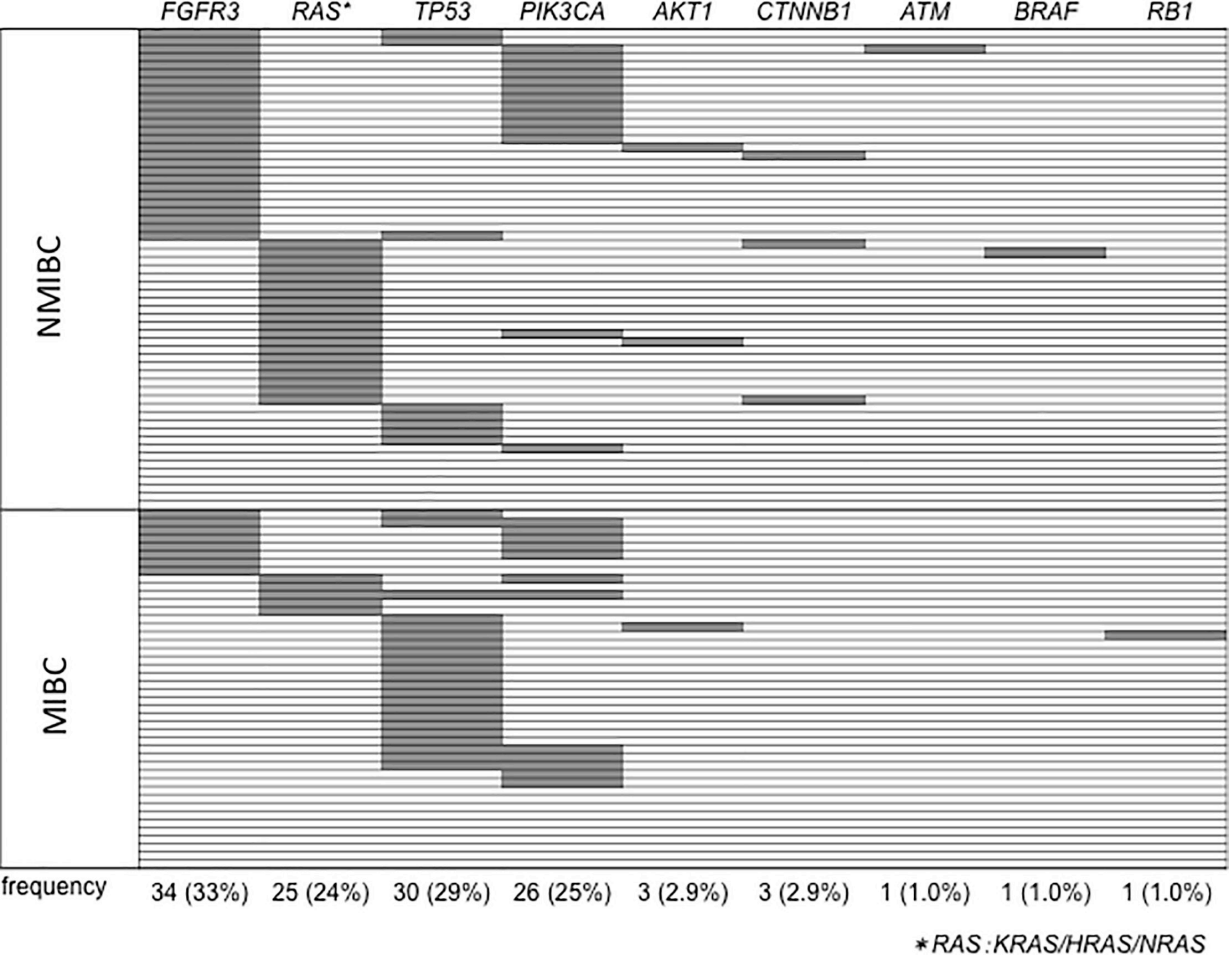
The frequency of somatic mutations. Upper half shows the results of NMIBC patients, and lower half shows that of MIBC patients. We analyzed 50 cancer-related genes with Ion Ampli Seq Cancer Hotspot Panel v2 and listed the genes with mutations. Each horizontal column indicates a patient. Grey columns indicate the presence of mutation.

Mutation analysis showed that *FGFR3*, *TP53*, *PIK3CA*, *RAS* (*HRAS*, *KRAS*, or *NRAS*), *AKT1*, *CTNNB1*, *ATM*, *BRAF*, and *RB1* mutations were present in at least one or more patients (Fig 1 and S1 Table). There were no mutations in the remaining 39 genes. The prevalence of *FGFR3* mutation was 33%, followed by *TP53* (29%), *PIK3CA* (25%), and *RA*S (24%) mutation. *FGFR3* mutation was mutually exclusive with *RAS* mutation (*p* < 0.01; *RAS* mutation included *HRAS*, *KRAS*, *and NRAS*) and *TP53* mutation (*p* = 0.02), but co-existent with *PIK3CA* mutation (*p* < 0.01). *TP53* mutation and *RAS* mutation were also exclusive (*p* < 0.01). Other mutations were not correlated with each other. The prevalence of mutations in *FGFR3* or *RAS* was higher in NMIBC than in MIBC (*FGFR3* was 51% and 25%, while *RAS* was 27% and 9.4% in NMIBC and MIBC, respectively).

Table 1 shows the frequencies of *TP53* codon 72 polymorphism. Three genotypes, Arg/Arg, Arg/Pro, and Pro/Pro, were found in 37% (38/103), 42% (43/103), and 21% (22/103) of the patients, respectively. The results fit the Hardy-Weinberg equilibrium. As shown in Table 1, clinical characteristics, including tumor grade, tumor stage, or smoking status, were not significantly different among the patients. In contrast, there was a significant difference in the prevalence of mutations in *FGFR3* and *PIK3CA* (*p* < 0.01 and *p* = 0.04, respectively) among *TP53* codon 72 polymorphisms but not in *RAS* and *TP53*. Patients with Pro/Pro had significantly lower *FGFR3* mutation rates and patients with Arg/Arg had higher *PIK3CA* mutation rates, as evaluated with adjusted residuals.

**Table 1:** Patient characteristics and prevalence of somatic mutations with respect to the *TP53* codon 72 polymorphism.

		Total, n	Arg/Arg n (%)	Arg/Pro n (%)	Pro/Pro n (%)	*p*-value
*TP53* codon 72 polymorphism		103	38 (37)	43 (42)	22 (21)	
Age	Median	71	67	73	71	0.24**
(year)	(range)	(39–87)	(41–86)	(39–87)	(60–87)	
Sex	male	85	34 (86)	33 (77)	18 (82)	0.32***
	female	18	4 (14)	10 (23)	4 (18)	
Clinical	NMIBC(≦T1)	59	21 (55)	28 (65)	10 (45)	0.30***
Stage	MIBC(≧T2)	44	17 (45)	15 (35)	12 (55)	
Grade	Low	33	12 (32)	16 (37)	5 (23)	0.49***
	High	70	26 (68)	27 (63)	17 (77)	
Smoking	non-smoker	34	11 (30)	15 (35)	8 (36)	0.81***
status	smoker	62	24 (63)	24 (56)	14 (64)	
	unknown	7	3 (8)	4 (9)	0	
Somatic	*FGFR3*	34	16 (42)	17 (40)	1 (4.5)	< 0.01***
mutation	RAS*	25	10 (26)	6 (14)	9 (41)	0.053***
	*PIK3CA*	26	15 (39)	8 (19)	3 (14)	0.04***
	*TP53*	30	9 (24)	15 (35)	6 (27)	0.53***

*RAS: KRAS/HRAS/NRAS

**: Kruskal-Wallis test

***: chi-square test

### Influence of Arg allele of *TP53* codon 72 polymorphism on *FGFR3* or *RAS* mutations in NMIBC

We further analyzed the allele of *TP53* polymorphism related to the prevalence of somatic mutations in bladder cancer-related genes (Table 2). When both NMIBC and MIBC patients were analyzed and divided into two groups as having Arg allele (Arg/Arg or Arg/Pro) and not having Arg allele (Pro/Pro), the prevalence of mutations in *FGFR3* was significantly higher in patients with the Arg allele (41% vs 4.5%, *p* < 0.01). In contrast, the prevalence of mutations in *RAS* was higher in patients without the Arg allele (41% vs 20%, *p* = 0.04). The prevalence of mutations in *PIK3CA* and *TP53* was not different between these two groups. When the patients were stratified into groups as having Pro allele (Pro/Pro or Arg/Pro) and not having Pro allele (Arg/Arg), only the prevalence of mutations in *PIK3CA* was higher in patients without the Pro allele (39% vs 17%, *p* = 0.01).

**Table 2:** Frequency of somatic mutations divided according to the clinical stage in *TP53* codon 72 polymorphism.

		*TP53* codon72 polymorphism					
	Somatic Mutation	with Arg allele, n (%)	without Arg allele, n (%)	p** value	without Pro allele, n (%)	with Pro allele, n(%)	p** value
All cases	N	81	22		38	65	
	*FGFR3*	33 (41)	1 (4.5)	**< 0.01**	16 (42)	18 (28)	0.13
	RAS*	16 (20)	9 (41)	**0.04**	10 (26)	15 (23)	0.71
	*PIK3CA*	23 (28)	3 (14)	0.16	15 (39)	11 (17)	**0.01**
	*TP53*	24 (30)	6 (27)	0.83	9 (24)	21 (32)	0.35
NMIBC	n	49	10		21	38	
	*FGFR3*	25 (51)	1 (10)	**0.02**	11 (52)	15 (19)	0.34
	RAS*	13 (27)	7 (70)	**< 0.01**	7 (33)	13 (34)	0.95
	*PIK3CA*	13 (27)	1 (10)	0.26	8 (38)	6 (16)	0.054
	*TP53*	7 (14)	1 (10)	0.72	1 (4.8)	7 (18)	0.14
MIBC	n	32	12		17	27	
	*FGFR3*	8 (25)	0 (0)	0.06	5 (29)	3 (11)	0.13
	RAS*	3 (9.4)	2 (17)	0.50	3 (18)	2 (7.4)	0.30
	*PIK3CA*	10 (31)	2 (17)	0.33	7 (41)	5 (19)	0.10
	*TP53*	17 (53)	5 (42)	0.50	8 (47)	14 (52)	0.76

*RAS: KRAS/HRAS/NRAS

**: chi-square test

Because mutations in *FGFR3* and *RAS* were more frequently detected in NMIBC, the prevalence of *TP53* codon 72 polymorphism and somatic mutations were separately analyzed in NMIBC and MIBC. In NMIBC, the prevalence of mutations in *FGFR3* was higher in patients with the Arg allele (51% vs 10%, *p* = 0.02) and the prevalence of *RAS* mutation was significantly higher in patients without the Arg allele (70% vs 27%, *p* < 0.01). In MIBC, no significant differences were identified between *TP53* codon 72 polymorphism and somatic mutations.

### *TP53* codon 72 polymorphism could affect the expression of *FGFR1* and *FGFR3*

Since fibroblast growth factor (FGF)-FGF receptor (FGFR) signaling pathways are activated not only through FGFR gene mutation, but also with overexpression of FGF-FGFR related genes [2], the expression of all 22 subclasses of FGF (*FGF1-FGF14*, *FGF16-FGF23*) and four subclasses of FGFR (*FGFR1-FGFR4*) was determined. The association of *TP53* codon 72 polymorphism with the expression of these genes was analyzed (S2 Table). Significant differences in the expression of *FGFR1* and *FGFR3* (*p* = 0.02 and *p* = 0.04, respectively) were observed, with no significant difference in the expression of other FGFs and FGFRs (S3 Table). In detail, comparing patients with Arg/Pro vs Pro/Pro, *FGFR1* expression was significantly higher in patients with Pro/Pro, and *FGFR3* expression was higher in patients with Arg/Pro (p* = 0.010 and p* = 0.015, respectively). Comparing patients with Arg/Arg vs Pro/Pro, *FGFR1* expression tended to be higher in patients with Pro/Pro, *FGFR3* expression was not significantly different (p* = 0.020 and p* = 0.38, respectively). Comparing patients with Arg/Arg vs Arg/Pro, neither *FGFR1* nor *FGFR3* was significantly different (p* = 0.73 and p* = 0.64, respectively). *: *p-*value was evaluated with Mann-Whitney’s U test with Bonferroni correction.

We further analyzed the allele of *TP53* polymorphisms related to the expression of *FGFR1* and *FGFR3*. As shown in Table 3, when both NMIBC and MIBC patients were analyzed, *FGFR3* expression was higher in patients with the Arg allele (FDR-*p* = 0.02). In NMIBC patients, *FGFR3* expression was higher in patients with the Arg allele (FDR-*p* = 0.02). *FGFR1* expression was higher in patients without the Arg allele (FDR-*p* < 0.01). However, in MIBC patients, no significant differences were identified between *TP53* codon 72 polymorphism and expression of FGFRs.

**Table 3:** *FGFR1/3* mRNA expression with respect to the *TP53* codon 72 polymorphism.

			*FGFR1* expression		*FGFR3* expression	
Clinical stage	*TP53* codon72 polymorphism	total, n	mean +/- SD	FDR-p* value	mean +/- SD	FDR-p* value
All cases	with Arg allele	81	1.18 +/- 8.11	0.14	82.85 +/- 128.78	**0.02**
	without Arg allele	22	2.84 +/- 8.64		35.66 +/- 77.39	
	without Pro allele	38	1.28 +/- 8.99	0.66	63.36 +/- 97.28	1
	with Pro allele	65	1.29 +/- 7.91		54.14 +/- 135.48	
NMIBC	with Arg allele	49	0.90 +/- 2.17	**< 0.01**	120.07 +/- 144.98	**0.02**
	without Arg allele	10	2.05 +/- 2.50		50.83 +/- 28.21	
	without Pro allele	21	0.90 +/- 2.32	0.79	152.85 +/- 107.17	1
	with Pro allele	38	1.01 +/- 2.25		79.26 +/- 100.57	
MIBC	with Arg allele	32	2.10 +/- 12.26	1	40.01 +/- 83.26	1
	without Arg allele	12	4.33 +/- 10.67		21.36 +/- 103.68	
	without Pro allele	17	1.90 +/- 13.11	1	34.67 +/- 79.68	1
	with Pro allele	27	3.26 +/- 11.15		25.71 +/- 94.50	

*: Wald test with Benjamini-Hochberg multiple test correction

### Combination of smoking status and *TP53* codon 72 polymorphism could affect somatic mutations in NMIBC

**Fig 2:**
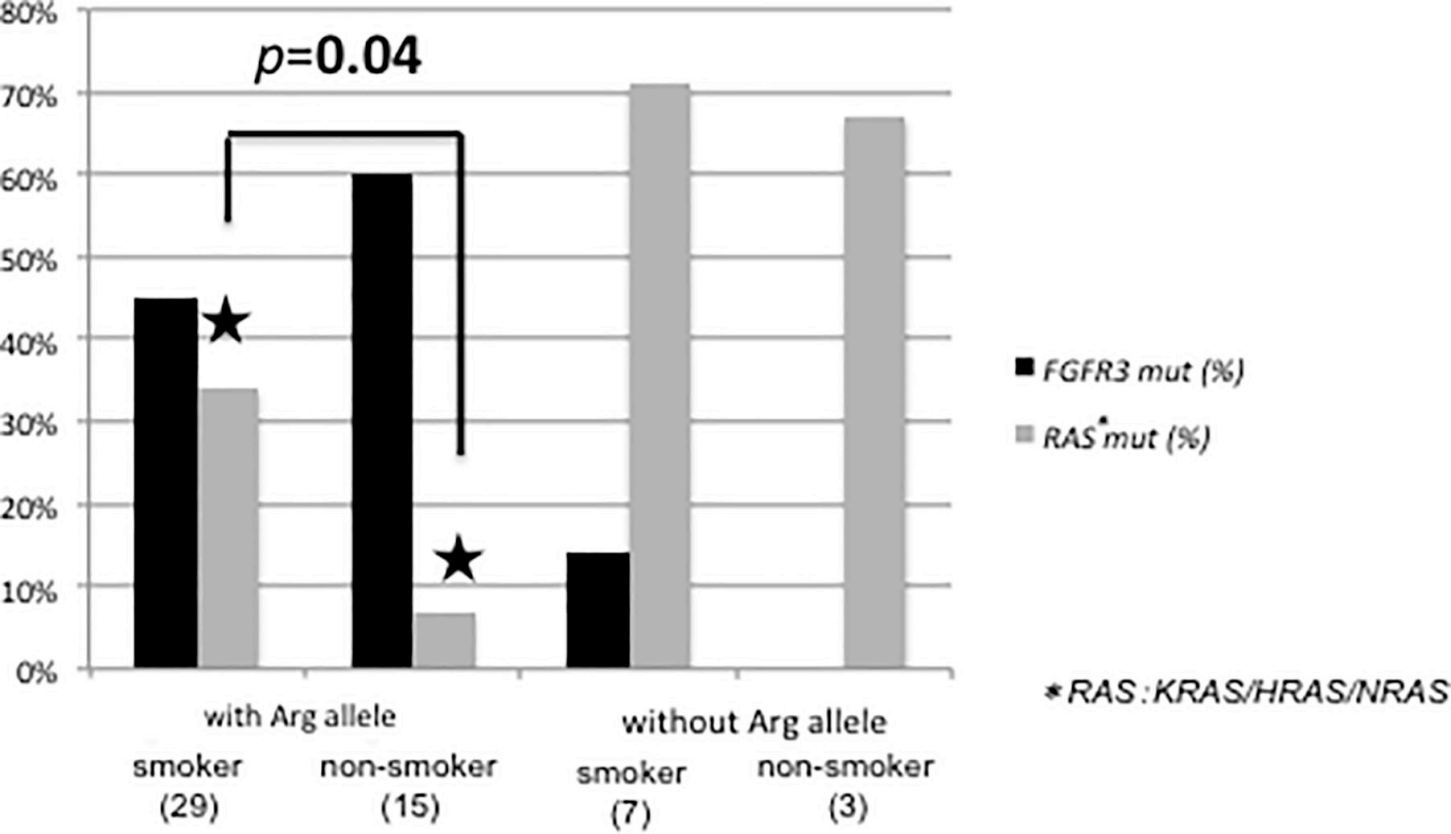
Prevalence of mutations in *FGFR3* and *RAS* in *TP53* codon 72 polymorphism and smoking status of NMIBC patients. Black bar and gray bar indicate the frequencies of *FGFR3* and *RAS* mutation, respectively. The number in parentheses indicates the patients’ number.

Since smoking status has been associated with somatic mutation [27], we analyzed the frequency of *FGFR3* or *RAS* mutations in *TP53* codon 72 polymorphism and smoking status. Among NMIBC patients with the Arg allele, the prevalence of *RAS* mutation was significantly higher in smokers than in non-smokers (34% (10/29) vs 6.7% (1/15), *p* = 0.04). In contrast, among NMIBC patients without the Arg allele, there was no significant difference between the prevalence of *RAS* mutations and smoking status (smokers 71% (5/7) and non-smokers 67% (2/3); *p* = 0.88, Fig 2). There was no significant association between smoking status and *FGFR3* or *RAS* mutations in *TP53* codon 72 polymorphism among MIBC patients.

## Discussion

In this study, we show that germline *TP53* codon 72 polymorphism could affect *FGFR3* and *RAS* mutations in NMIBC. Although there are some reports on the association between *TP53* codon 72 polymorphism and *TP53* mutation in NSCLC [23, 24], to our knowledge, there is no report on the relationship between *TP53* codon 72 polymorphism and somatic mutations, other than *TP53* mutation. In our dataset, no association between *TP53* mutation and *TP53* codon 72 polymorphism was observed (Tables 1 and 2). However, patients with the Arg allele were associated with *FGFR3* mutation and patients without the Arg allele were associated with *RAS* mutation in NMIBC (Table 2). To the best of our knowledge, this is the first report clarifying the relationship between germline *TP53* codon 72 polymorphism and somatic mutations in bladder cancer. Several studies have identified that germline polymorphisms are associated with specific somatic mutations [18–22]. Our results also imply that germline background could affect the specific somatic mutations in bladder cancer.

The FGF-FGFR signaling pathway is composed of several subtypes of FGFs and FGFRs. Some studies have focused on activating *FGFR1* and *FGFR3* in bladder cancer [28, 29]; however, other FGFRs and FGF-ligands were not studied. Although the activation and overexpression of *FGFR3* have been reported [30], the relationship between other FGF-FGFR subtypes and their expression status have not been documented. We show that the *TP53* codon 72 polymorphism is also associated with the expression of *FGFR1* and *FGFR3*. Expression of *FGFR1* was higher in patients without the Arg allele, while expression of *FGFR3* was higher in patients with the Arg allele. Because *FGFR3* mutation activates point mutations [2], its expression was higher in patients with the Arg allele who show higher frequency of the *FGFR3* mutation. On the other hand, the reason for higher expression of *FGFR1* in patients without the Arg allele is unclear. A previous study showed that silencing *FGFR1* expression using small interfering RNA was effective in elevating *FGFR3* expression and tumor supportive activity, suggesting that *FGFR1* and *FGFR3* have an inverse relationship [31].

Several studies and the cohort presented here show that *FGFR3* and *RAS* mutations are mutually exclusive events in bladder cancer [10, 27]. The mutual exclusivity of *FGFR3* and *RAS* gene mutations is thought to reflect activation of the same pathway. The oncogenic role of activated *FGFR3* is mediated by the activation of mitogen-activated protein kinase through the RAS signaling pathway [32]. *FGFR3* mutations are strongly associated with low-grade and low stage bladder cancer, with lower frequency of recurrence [33]. Unlike *FGFR3* mutations, no relation of *RAS* mutational pattern with tumor grade and stage has been found [10]. *FGFR3* mutation was seen in about 70% and *RAS* mutation in about 20% of low-grade non-invasive papillary tumors [27]. However, whether *FGFR3* or *RAS* mutation is selected in NMIBC is unclear. Our results suggest that *TP53* codon 72 polymorphism contributes to the selection of somatic mutations in NMIBC.

Several environmental or habitual factors, including smoking and inflammation, have been associated with bladder carcinogenesis. The dataset presented here suggests that smoking status could affect the somatic mutations in NMIBC, based on *TP53* codon 72 polymorphism. In patients with the Arg allele, *FGFR3* mutation was higher than *RAS* mutation. When divided into smoking status, non-smokers with the Arg allele rarely had *RAS* mutation. On the other hand, patients without the Arg allele had *RAS* mutation, regardless of smoking status. Previous reports show that smoking is associated with somatic mutations. In NSCLC, *EGFR* mutations are more frequently found in non-smokers [34, 35]. Unlike *EGFR* mutations, most *KRAS* mutated NSCLC patients are former or current smokers [36–38]. In bladder cancer, a few studies have examined the association between smoking and somatic mutations. The cancer genome atlas (TCGA) data shows no statistically significant association between smoking status and somatic mutations [39]. Pandith et al. reported that *FGFR3* and *RAS* mutations were higher in smokers, but no significant association was found [27]. TCGA analyzed only MIBC patients [39], whereas Pandith et al. analyzed both MIBC and NMIBC patients [27]. These discrepancies were probably due to analysis of different clinical background factors in patients. Nonetheless, these results suggest that different types of bladder cancer have specific mutations depending on the *TP53* codon 72 polymorphism and smoking status.

**Fig 3:**
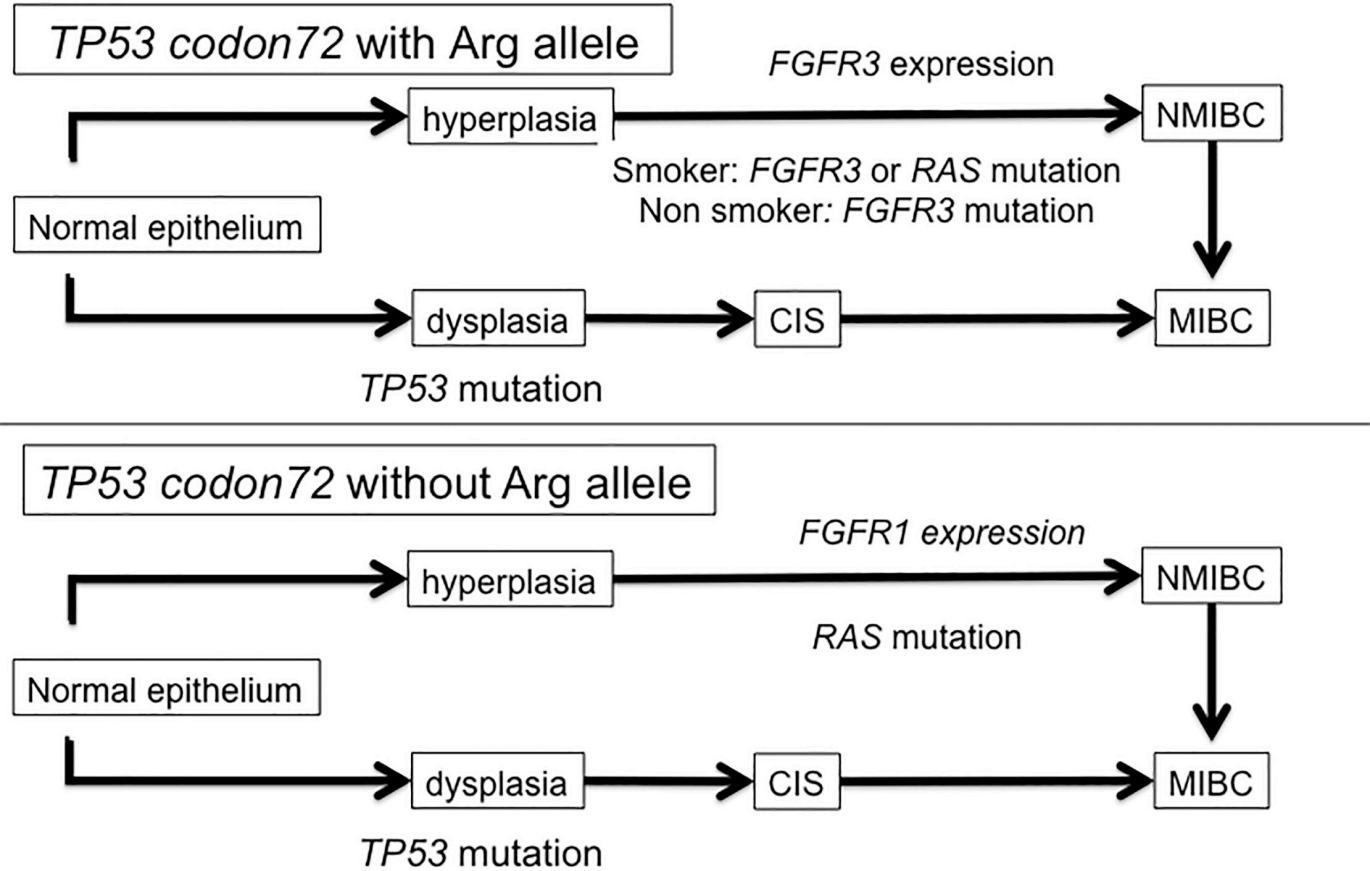
Proposed mechanism of two-pathway model in bladder carcinogenesis considering *TP53* polymorphism and smoking status.

Based on the previous bladder carcinogenesis model [7] and our results, we propose a new carcinogenesis model in NMIBC (Fig 3). *FGFR3* and *RAS* mutation could be affected by the *TP53* codon 72 polymorphism and smoking status in NMIBC. Non-smokers with the Arg allele show *FGFR3* mutation and smokers with the Arg allele show either *FGFR3* or *RAS* mutation. Patients without the Arg allele show *RAS* mutation regardless of smoking status. Moreover, germline *TP53* polymorphisms could affect the expression of *FGFR1* and *FGFR3* in NMIBC. *FGFR3* expression was higher in patients with the Arg allele, and *FGFR1* expression was higher in patients without the Arg allele. Taken together these results suggest that *TP53* codon 72 polymorphism and smoking status could affect somatic mutations and FGF-FGFR signaling in bladder carcinogenesis.

Several reports have shown that *TP53* codon 72 polymorphism is a response associated with chemotherapy or radiotherapy [40, 41]. The mechanisms underlying the influence of the *TP53* codon 72 genotype on anticancer treatment response are still unknown. Although our data show the relationship between *TP53* codon 72 polymorphism and mutations in FGF-FGFR gene expression, our results cannot explain the mechanism or the difference in treatment response among *TP53* codon 72 polymorphism.

Although our study revealed associations between *TP53* codon 72 polymorphisms and somatic mutations in bladder cancer, it was limited by the relatively small sample size. Additionally, the cohort consisted of Japanese bladder cancer patients only; there is still a lack of clarity in the underlying mechanism, and we could not identify a treatment strategy around the *TP53* codon 72 polymorphism. Therefore, studies involving larger cohorts and other ethnicities are needed to confirm our results. Nonetheless, our results contribute to the elucidation of the mechanism of bladder carcinogenesis. Further clarification regarding the relation between bladder carcinogenesis and genetic background will aid the development of bladder cancer therapy.

In conclusion, *TP53* codon 72 polymorphism is associated with *FGFR3* or *RAS* mutation in NMIBC, suggesting that host germline could affect the selection for somatic mutation type in bladder carcinogenesis.

## Supporting information

S1 Table Patients’ metadata and mutation status. (XLSX)

S2 Table FGF-FGFR expression values. (XLSX)

S3 Table Comparison between expression of FGF-FGFR signaling pathway genes and *TP53* codon 72 polymorphism. (XLSX)
